# Management of Macular Pre-Retinal Subhyaloid hemorrhage by Nd:Yag laser hyaloidotomy

**Published:** 2014

**Authors:** Faisal Murtaza, Syed Fawad Rizvi, Syeda Aisha Bokhari, Zeeshan Kamil

**Affiliations:** 1 Dr. Faisal Murtaza, MBBS, MCPS, FCPS, FRCS, Consultant Ophthalmologist, L.R.B.T Free Base Eye Hospital, Korangi no. 2 ½, Karachi, Pakistan.; 2 Dr. Syed Fawad Rizvi, MBBS, MCPS, FCPS, Chief Consultant Ophthalmologist, L.R.B.T Free Base Eye Hospital, Korangi no. 2 ½, Karachi, Pakistan.; 3 Dr. Syeda Aisha Bokhari, MBBS, FCPS, Ophthalmologist, L.R.B.T Free Base Eye Hospital, Korangi no. 2 ½, Karachi, Pakistan.; 4 Dr. Zeeshan Kamil, MBBS, MCPS, FCPS, Ophthalmologist, L.R.B.T Free Base Eye Hospital, Korangi no. 2 ½, Karachi, Pakistan.

**Keywords:** Nd-YAG laser, Subhyaloid Hemorrhage

## Abstract

***Objective: ***To evaluate the efficacy, visual outcome and complication following Nd:Yag laser hyaloidtomy for subhyaloid hemorrhage.

***Methods: ***This interventional case series was managed at LRBT, Free Base Eye Hospital Karachi from January 2010 to December 2010. It included 30 eyes of 30 patients with subhyaloid hemorrhage due to different causes which underwent Nd: Yag laser sublyaloidotomy

***Results: ***Out of thirty patients, eighteen (60%) were male and twelve (40%) were females. Mean age was 32.57 years. Males pre-dominated the study. Pre laser visual acuity was between counting finger at one meter in 22 patients (73.33%) and between counting finger one meter to hand movement in 8 patients (26.66%). Vision improved to 6/6 in 10 patients (33%), 6/9 – 6/12 in 17 patients (56.66%) and between 6/24 – 6/60 in 3 patients (9.99%) at the end of follow up. Complications were persistent vitreous hemorrhage in one (3.33%) patient, failed drainage in one (3.33%) patient and metamorphopsia in one (3.33%) patient.

***Conclusion: ***Nd: Yag laser hyloidotomy is an excellent technique for management of Subhyaloid hemorrhage with early visual recovery provided there is no macular pathology.

## INTRODUCTION

Pre-retinal Subhyaloid hemorrhage or sub internal limiting membrane hemorrhage may occur after retinal vascular rupture associated with physical exertion, increased venous pressure (valsalva maneuvere) or various retinal vascular disorders. It is associated with proliferative diabetic retinopathy, retinal artery macro aneurysm, trauma,^1^ age-related maculopathy, hematological disorders such as leukemia and chemotherapy induced pancytopenia, following laser in situ keratomileusis because of rapid release of the microkeratotome vacum pressure or after retinal vascular rupture associated with physical exertion (valsalva retinopathy), Terson’s syndrome, Purtscher’s syndrome.^2^ Visual acuity is often profoundly reduced but spontaneous clearance usually occurs within few months.^3^

Nd: yag laser (neodymium yag laser) is commonly used for a variety of anterior segment procedures particularly iridotomy and posterior capsulotomy. Posterior segment applications of the Nd: yag laser have generally been limited to transaction of vitreous membranes in selected cases of diabetic tractional retinal detachment, sickle cell retinopathy and complicated retinal detachment.^4^^,^^5^ Subhyaloid hemorrhage can drain internally with 3 ports pars plana vitrectomy and internal limiting membrane peeling as well as by yag laser hyloidotomy.^6^^,^^7^ Nd: yag Laser provides an alternate safe method of internal drainage in the management of macular subhyaloid hemorrhage.^8^ Our objective was to evaluate the efficacy, visual outcome and complication following Nd:Yag laser hyaloidtomy for subhyaloid hemorrhage.

## METHODS

This quasi experimental interventional study was carried out at LRBT, Free Base Eye Hospital, Korangi, Karachi from January 2010 to December 2010 and included 30 eyes of 30 patients. Study was conducted with the approval of hospital ethical committee. All surgeries were done by two surgeons (FM and SFR). The data acquisition was performed by two investigators (SAB and ZK) independently of the surgeons. A performa was designed to record relevant information which included name, age, sex, occupation, with presenting complaints in chronological order, special attention was focused on nature of visual loss, sudden or gradual. History of systemic disease like diabetes, hypertension, trauma, previous ocular surgery, family history and bleeding disorders was collected. Complete ocular examination was performed including vision for both distance and near, pupillary examination, amsler grid test, slit lamp examination of anterior segment to look for any sign of trauma, iris neovascularization if any. Intraocular pressure with applanation tonometry was recorded before dilatation of pupil. Pupil was dilated with tropicamide 1%. Fundus examination was carried out by an indirect ophthalmoscope and 78 diopter noncontact lens slit lamp biomicroscopy. Configuration of Subhyaloid hemorrhage with size measured in disc diameter & location were noted, rest of the fundus was examined in detail to identify diabetic retinopathy hypertensive retinopathy, central retinal vein occlusion, branch retinal vein occlusion, posterior vitreous detachment and retinal breaks. Fundus photographs were also taken. Systemic investigations include recording of blood pressure, pulse, complete blood count, blood sugar, bleeding time, clotting time.

***Procedure: ***When the patients had been diagnosed with subhyaloid heamorhage, they were informed of their diagnosis and explained that there was a slight possibility that their condition would resolve spontaneously. They were then informed of the study to be conducted and were invited to participate. Patients were enrolled in the study when they had given their informed consent.

**Fig.1:**
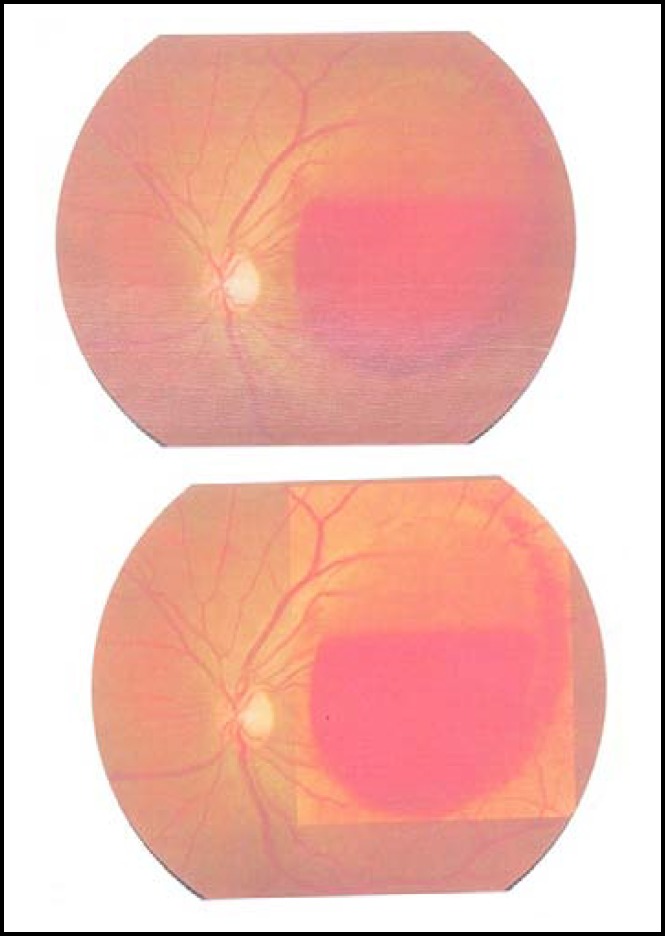
Fundus photograph. Pre-yag hyaloidotomy

**Fig.2:**
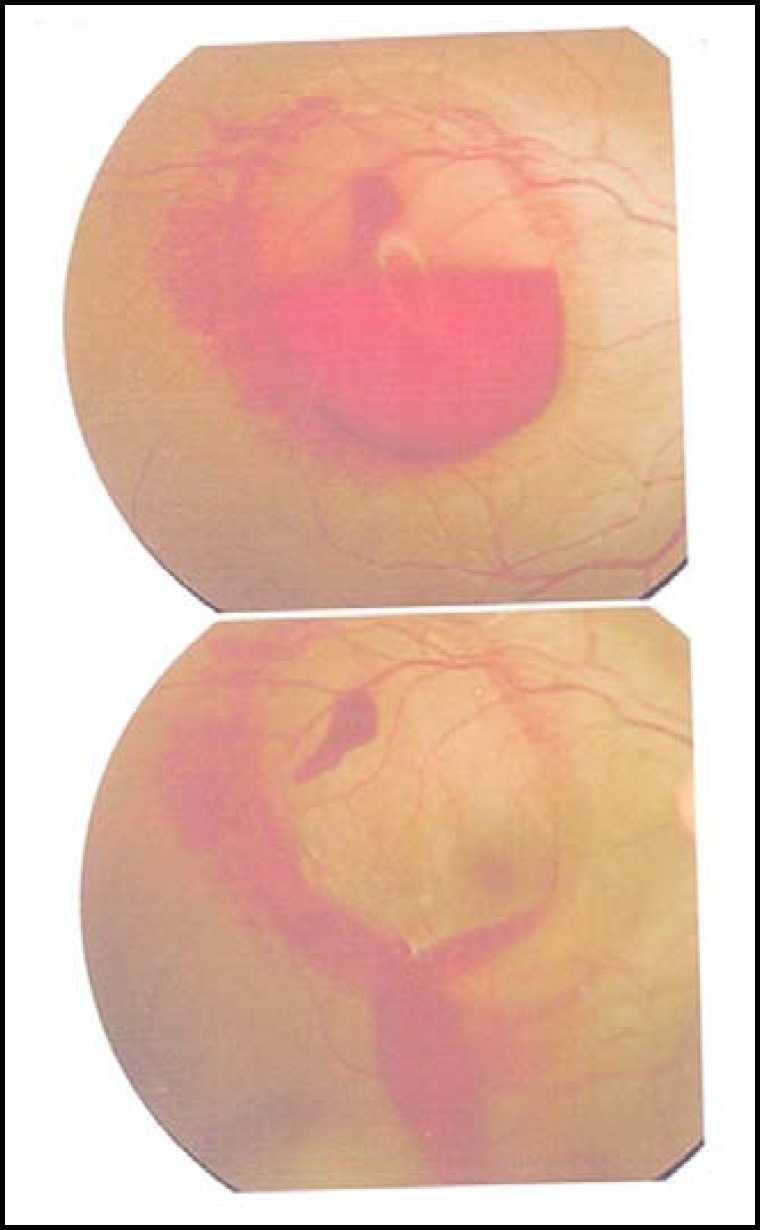
Fundus photograph. Post-yag hyaloidotomy

All laser treatments were performed under topical anaesthesia with 0.5% proparacaine (alcaine). Mainster area centralis (Ocular) lens was used. Laser was operated at Q-switch fundamental mode at energy level between 1.9 – 11.5 mj from low to high energy level fire at the anterior surface and inferior margin of the hemorrhage away from the fovea and 2 to 3 openings were made until a rapid stream of blood was seen trapped into the vitreous cavity.

Post procedure patients were given topical non-steroidal anti-inflammatory agents 4 times a day for 7 days. Patients were followed after one week, 3 weeks, 6 weeks. On each follow up visit, visual acuity for distance and near, anterior segment examination and fundus examinations were recorded on performa.

## RESULTS

This study includes thirty eyes of thirty patients out of which eighteen (60%) were male and twelve (40%) were females. Age range was 15-60 years, 8 patients 26.66% were between 15-30 years, 12 patients 40% were between 30-45 years and 10 patients 33.33% were between 45-60 years. Etiology of Subhyaloid hemorrhage were noted as diabetes in 15 (50%) cases, valsalva retinopathy in 6 (20%) cases, trauma in 3 (10%) cases, idiopathic 2 (6.66%) cases, retinal artery macroaneurysm in 2 (6.66%) patients, blood dyscrasia in 1 (3.33%) patient & neovessels on disc secondary to branch retinal vein occlusion in 1 (3.33%) patient. 

Presenting complaint was reduced visual acuity in 28 (93.335%) patients & object appears red in 2 (6.66%) patients. Size of Subhyaloid hemorrhage was 3 DD (disc diameter) in eight (26.66%) cases, 4 to 5 DD in 12 (40%) cases and 6 to 12 DD in 10 (33.335) cases. Pre laser visual acuity was between counting finger near face to 1meter in 22 patients (73.33%) and between counting finger 1meter to hand movement in 8 patients (26.66%) were improved to 6/6 in 10 patient 33%, 6/9 – 6/12 in 17 patient (56.66%) and between 6/24 – 6/60 in 3 patients (9.99%) at the end of follow up. Duration of absorption of haemorrhage was 6-23 days. Success rate was seen in 27 patients (93.33%). There were few complications noted. There was no damage to retina and choroid.

## DISCUSSION

Sub hyaloids heamorhage is defined as a localized detachment of vitreous from the retina caused by the accumulation of blood which can lead to sudden and severe loss of vision when it takes place in the macular area.^9^^-^^10^ One of the recent indications for the application of Nd-YAG laser in posterior segment is to treat pre macular subhyaloid heamorhage.^11^ In this study we performed posterior hyaloidotomy using Nd-YAG laser in 30 patients with pre macular subhyaloid heamorhage. The cause of heamorhage included diabetic retinopathy, valsalva retinopathy, trauma, retinal artery aneurysm blood dyscrasia and branch retinal vein occlusion. In 29 out of 30 cases, the trapped blood was released into the vitreous and resorbed within 6-23 days (mean 14.5 days). Mehdi Ahmedabadi reported resorption of blood within 9 days^11^, whereas, Celebi S observed it within one week.^12^ In another study Mehdi Ahmedabadi reported resorption in 14.5 days.^14^ Similar results were also observed by Rennie.^15^

All patients showed improvement in their visual acuity within 2 weeks of the procedure. Post-operative best corrected visual acuity reached 6/6 in 10 patients (33%), 6/9-6/12 in 17 patients (56.66%) and 6/24-6/60 in 3 patients (9.99%).

Rennie evaluated 10 patients with pre macular subhyaloid heamorhage of different etiologies. Nd-YAG laser hyaloidotomy was performed in 6 patients while 4 patients were managed conservatively. Visual acuity improved to 6/9 in 4 out of 6 patients (66.66%).^15^

In the study conducted by Khan ^16^, a total of 11out of 12 patients opted for Nd-YAG laser treatment. Visual acuity improved in all 11 patients. In another study conducted by Durukan 14 out of 16 patients achieved visual acuity of 20/20 in 1 week and the remaining 2 patients achieved 20/20 level in 1 month.^17^

The procedure was successful in 27 out of 30 patients (93.33%) in this study. In the pilot study conducted by Mehdi Ahmedabadi^11^, hyaloidotomy was successful in all (100%) patients. In another study carried by Mehdi Ahemdabadi 12 out of 14 patients (84.7%) in the laser group were successful^14^.

Ulbig studied 21 patients with premacular subhyaloid heamorhage of different etiologies. Hyaloidotomy was successful in 16 (76.2%) of their patients.^12^ Gabel evaluated 3 patients with premacular subhyaloid heamorhage. Nd-YAG laser hyaloidotomy was successful in all cases.^1^

Complications observed in the study include persistent vitreous heamorhage in one patient (3.33%), failed drainage of sub macular heamorhage in one patient (3.33%) and metamorphopsia in one patient (3.33%). Mehdi Ahmedabadi study showed failed drainage in 2 out of 21 patients (9.52%) due to clot heamorhage for which they underwent additional vitrectomy. Epiretinal membrane formation was seen in one case (4.76%) during follow up.^14^

Ulbig reported macular hole in 1 out of 21 eyes (4.76%) and retinal detachment from a retinal break in a myopic patient (4.76%).^13^

## CONCLUSION

Considering the results of this study, it can be concluded, that Nd-YAG laser hyalodotomy is a simple safe and inexpensive out-patient procedure. This technique can prevent long-term entrapment of blood and its adverse effects on macula including potential permanent visual loss. Further controlled clinical trials are required to compare this treatment with other modalities.

## Authors’ contribution:

**FM and SFR** performed the surgeries.

**SAB and ZK **did data acquisition and manuscript writing.
